# Novel Non-invasive Transcranial Electrical Stimulation for Parkinson’s Disease

**DOI:** 10.3389/fnagi.2022.880897

**Published:** 2022-04-12

**Authors:** Rui Ni, Ye Yuan, Li Yang, Qiujian Meng, Ying Zhu, Yiya Zhong, Zhenqian Cao, Shengzhao Zhang, Wenjun Yao, Daping Lv, Xin Chen, Xianwen Chen, Junjie Bu

**Affiliations:** ^1^Department of Intelligent Medical Engineering, School of Biomedical Engineering, Anhui Medical University, Hefei, China; ^2^Department of Life Sciences, Imperial College London, London, United Kingdom; ^3^Department of Radiology, The Second Affiliated Hospital of Anhui Medical University, Hefei, China; ^4^Department of Neurology, The Fourth Affiliated Hospital of Anhui Medical University, Hefei, China; ^5^Department of Neurology, The First Affiliated Hospital of Anhui Medical University, Hefei, China; ^6^Department of Neurosurgery, The Fourth Affiliated Hospital of Anhui Medical University, Hefei, China

**Keywords:** transcranial electrical stimulation (tES), Parkinson’s disease, closed loop stimulation, neuromodulation, non-invasive treatment

## Abstract

Conventional transcranial electrical stimulation (tES) is a non-invasive method to modulate brain activity and has been extensively used in the treatment of Parkinson’s disease (PD). Despite promising prospects, the efficacy of conventional tES in PD treatment is highly variable across different studies. Therefore, many have tried to optimize tES for an improved therapeutic efficacy by developing novel tES intervention strategies. Until now, these novel clinical interventions have not been discussed or reviewed in the context of PD therapy. In this review, we focused on the efficacy of these novel strategies in PD mitigation, classified them into three categories based on their distinct technical approach to circumvent conventional tES problems. The first category has novel stimulation modes to target different modulating mechanisms, expanding the rang of stimulation choices hence enabling the ability to modulate complex brain circuit or functional networks. The second category applies tES as a supplementary intervention for PD hence amplifies neurological or behavioral improvements. Lastly, the closed loop tES stimulation can provide self-adaptive individualized stimulation, which enables a more specialized intervention. In summary, these novel tES have validated potential in both alleviating PD symptoms and improving understanding of the pathophysiological mechanisms of PD. However, to assure wide clinical used of tES therapy for PD patients, further large-scale trials are required.

## Introduction

Parkinson’s disease (PD) is the second most common neurodegenerative disorder (Wirdefeldt et al., 2011). The diminishing number of pigmented dopaminergic neurons in the substantia nigra and the presence of Lewy bodies are the hallmarks of PD, resulting in both motor and cognitive impairments. The motor symptoms of PD typically include resting tremor, rigidity, and stoop posture, while the non-motor symptoms involve defects in patients’ memory and emotions, sometimes dementia (Beitz, 2014).

The most recent decades were marked by extensive research efforts in developing novel pharmacological and physical therapies to ameliorate symptoms of PD. Levodopa has been widely used in PD treatment and proved to be extremely effective in alleviating motor symptoms during early disease stages. Nevertheless, levodopa has fell short treating non-motor impairments, which are especially common during the late stages of PD (Fabbri et al., 2017). Furthermore, undesirable side effects and drug resistance accompanied by disease progression hindered levodopa efficacy (Nonnekes et al., 2016). Therefore, a pivotal objective of current tES studies is to develop potential therapeutic interventions to relieve both motor and non-motor symptoms.

Over the past decades various neuromodulation techniques have been trialed, including deep brain stimulation (DBS), transcranial magnetic stimulation (TMS), and transcranial electrical stimulation (tES). DBS has now been clinically tested in late-stage PD. However, DBS surgery involves implantation of electrodes into deep brain regions, with significant safety risk (Morishita et al., 2017). Non-invasive stimulation approaches such as TMS have the risk of activating facial muscles, which leads to unpleasant feelings and forestalls double blind clinical trials (Weber et al., 2014). In addition, TMS is relatively expensive and difficult to perform. In contrast, tES has been considered one of the most compelling PD interventions, not only because of its low cost but also because of its safety and ease of operation. tES is a non-invasive neuromodulation method by which a low-intensity current is applied over the subject’s scalp, hence facilitating or inhibiting abnormal neuron activity (Ganguly et al., 2020). tES includes transcranial direct current stimulation (tDCS), transcranial alternating current stimulation (tACS), transcranial pulsed current stimulation (tPCS), and transcranial random noise stimulation (tRNS). Since Conventional tDCS is one of the earliest non-invasive neuromodulation methods clinically tested in PD patients, while other tES methods either lack clinical testing in human PD populations, or may not have been studied in the context of PD, therefore this review classify only tDCS that contains one target electrode (anode/cathode) and a return electrode (Nitsche and Paulus, 2000) as the conventional tES.

Although conventional tDCS has shown some encouraging results in alleviating both motor and non-motor symptoms (Boggio et al., 2006; Bueno et al., 2019), in practice, effects of conventional tES fluctuated across different studies (Doruk et al., 2014; Lau et al., 2019). In addition, tDCS is relatively less effective than other existing interventions for PD, and the improvements on symptoms bring by tDCS are highly dependent on the patients’ condition. Therefore, in order achieve a promoted efficacy among large population of PD patients to be fully advantageous over other interventions, further development of tDCS is required. Here we present three possible reasons behind such inconsistent reports, each corresponding to a novel tES approach that tries to overcome this constraint.

Conventional tES might modulate the neuronal pathways that are not the main drivers of disease pathology or specific symptoms. Recently, the view that distinct pathologic pathways might be responsible for different PD symptoms has emerged. For example, PD symptoms can be recognized by impaired functional brain networks, which involve multiple brain regions (Boord et al., 2017). However, conventional tES is not able to adapt these novel pathology hypotheses, since it can only activate or suppress the activity of one specific brain region. Thus, novel tES modes target at different pathological pathways have been developed.

Additionally, improvements in behavior solely resulting from tES are often too subtle for accurate recording (Swank et al., 2016; Lau et al., 2019), therefore, some suggested that the cooperation of tES with other interventions might synergize and amplify the therapeutic effect by simultaneous modulating multiple pathways. It is also tempting to question whether the effects of tES would interact with other stimulations, serving as an accelerator of modulating efficiency.

Besides, tES efficacy highly depends on the subjects’ condition and considerable discrepancies have been observed following identical stimulation inter or intra- subject. Therefore, the individualization of tES (e.g., closed-loop and brain-informed tES) is also gaining increasing interests owing to its “specialized to neuron response” characteristic. While conventional tDCS cannot automatically respond to these changes, novel stimulation approaches might give real-time individualized responses.

In this review, novel stimulation patterns are discussed and categorized into three groups: novel stimulation approaches, tES combination therapy with other interventions, and closed-loop stimulation. We will first discuss novel stimulation approaches with different modulation mechanisms toward PD. Then, the integrations of existing stimulation approaches with other types of PD intervention strategies will be introduced, and finally, we will analyze the closed-loop stimulation strategy that aims to achieve individualized self-adaptive parameters of stimulation. A detailed method and results of existing novel tES studies toward PD were summarized in Table 1.

**TABLE 1:** The detailed method and results of existing novel tES studies toward PD.

References	Stimulation method	Subjects (*n*)	Electrode montage	Targeted brain regions	Study design for tES	Results
**Novel stimulation modes**						
Dagan et al. (2018)	Multitarget, hd tDCS	20 PD with FOG	According to the 10–20 EEG system **anodes:** Cz, F3 **cathodes:** AF4, CP1, FC1, FC5	M1 Left DLPFC	Placebo-controlled, double blind, crossover (M1 tDCS, multitarget tDCS, sham) 20 minutes each session	FOG provoking test score↑ gait speed TUG performance↑ accuracy of Stroop test↑
Manor et al. (2021)	Multitarget, hd tDCS	77 PD with FOG	According to the 10–20 EEG system **anodes:** Cz, F3 **cathodes:** AF4, CP1, FC1, FC5	M1 Left DLPFC	Sham-controlled, double-blinded, randomized, 5 sessions per week, 2 weeks then 1 session per week, 5 weeks. 20 minutes each session	FOG provoking test:no improvements Likert global impression scale↑ daily living step counts↑. Mild to moderate PD patients revealed greater improvement and showed reduction in time completing FOG provoking test.
Shill et al. (2011)	tACS	23 PD	**Stimulation electrode:** frontal **reference electrodes:** bilateral mastoids	PFC	Sham-controlled, double-blinded, randomized, 77.5 Hz, 15 mA, 5 continuous stimulation sessions, 2 days break, followed by another 5-day session. 45 min per day.	Total off-medication UPDRS I-III: no improvements
Guerra et al. (2021)	β tACS and γ tACS	18 PD, 16 healthy subjects	According to the 10–20 EEG system **stimulation electrode:** centered over the First dorsal interosseus hotspot **reference electrode:** Pz	**M1**	Placebo-controlled, double blind, crossover (20 Hz, 70 Hz, sham in a session; 2 sessions on and off medication) 1 mA tACS. Each session = 3 blocks 15 s of task under each stimulation per block+ 4 min each stimulation together with TMS	Patients on medication: movement velocity↓ movement amplitude during β tACS compared to γ tACS. short interval intracortical inhibition (SICI)↓ in patients than HS
Krause et al. (2013)	tACS	10 PD–MCI, 10 healthy subjects	**Stimulation electrode:** above the M1 hot spot **reference electrode:** supraorbital region contralateral to the stimulation electrode.	**M1**	Placebo-controlled, double blind, crossover (10 Hz, 20 Hz, sham) 1 mA, 15 min each session	**20 Hz tACS** : finger tapping amplitude variation↓ CMC amplitude↓
Alon et al. (2012)	tPCS+ treadmill	10 PD with FOG	**Positive electrode:** right M1 **reference electrode:** left supraorbital area.	**M1**	Sham-controlled, double-blinded, monophasic, both pulse duration and interpulse intervals are 33.3 μs. week 1 20 min tPCS, week 2 20 min treadmill walking, third week 20 min tPCS.	**tPCS session:** gait speed and amplitude↑, steps backward↓ **treadmill session:** forward step length↑ **tPCS + treadmill session**: steps forward ↓
Monastero et al. (2020)	tRNS	10 PD-MCI	**Stimulation electrode:** left M1 **reference electrode:** contralateral shoulder.	M1	Placebo-controlled, double blind, randomized, crossover (active tRNS, sham) 1.5 mA randomly oscillating between 100–600 Hz 15 min each session	UPDRS total↑ UPDRS lateralized right and left score↑
Stephani et al. (2011)	tRNS	8 non-tremor-dominant idiopathic PD	**Stimulation electrode:** hot spot representing the largest reproducible MEP-response of the contralateral ADM, **reference electrode**:contralateral supraorbital region	M1	Double blind, ± 600 μA, maximum 640 Hz, 10 min	Cortical excitability↓
**tES combined with other interventions**						
Ferrucci et al. (2016)	hd tDCS with drug	9 PD with LIDs	According to EEG 10/20 system M1 tDCS: **stimulation electrode:** C3 and C4 **return electrode:** right deltoid muscle cerebellar tDCS: **stimulating electrode:** cerebellum **return electrode:** buccinator muscle	M1 cerebellar	Placebo-controlled, double blind, crossover (sham, cerebellar tDCS, M1 tDCS) 2 mA, 1 session per day, 5 continuous days, 20 min each session. Each condition at least 1 month separation.	UPDRS IV score↑
Chang et al. (2017)	tACS+ TMS	32 PD with FOG	According to EEG 10/20 system region for tACS **stimulation electrode:** F3 **reference electrode:** FP2	Left DLPFC	Placebo-controlled, double blind, randomized, 1 mA tACS, 1 session per day, 5 consecutive days, 20 min each session.	Executive function↑ in dual-mode PD subjects group compared to rTMS group
Guerra et al. (2020)	iTBS + tACS	16 PD, 16 healthy control	**Stimulating electrode:** centered over the first dorsal interosseus hotspot **reference electrode** :Pz	M1	Placebo-controlled, double blind, crossover (γtACS70 Hz,βtACS20 Hz, sham), 1 session per condition, approximately 3 mins and 30 s per session	iTBS-induced plasticity ↓in the iTBS-sham tACS session in patients, iTBS-γ tACS↑ abnormal plasticity, no long-lasting changes induced in M1 excitability
Kaski et al. (2014)	tDCS+ physical training	16 PD	**Stimulation electrode:** a region 10%–20% anterior to Cz as measured from the midline of the electrode **reference electrode:** inion	SMA	Sham-controlled, double blind, randomized,2 mA, physical training +tDCS and tDCS group, 15 min each group	tDCS+ physical training: gait speed↑ gait performance↑ TUG completing time↓ 6-minute walk test completing time↓ time needed to regain stability in pull test↓.
Conceição et al. (2021)	tDCS+ aerobic exercise	13 PD (only 7 analyzed)	According EGG 10–20 system **Anode**:F3/F4 (contralateral to most affected body side) **cathode:** FP2 or FP1 (contralateral to anode)	Personalized unilateral DLPFC	Placebo-controlled, double blind, cro20 min for each session,	Aerobic exercise + active-tDCS session: choice reaction time↓ step time variability↓ relative Hemoglobin levels in the stimulated hemisphere↓
Fernandez-Lago et al. (2017)	tDCS+ treadmill walking	18 PD	**Anode:** in the hotspot of the tibialis anterior muscle over motor cortex contralateral to the most affected body side **cathode:** supraorbital region contralateral to anode.	M1	Placebo-controlled, double blind, crossover (treadmill, treadmill+ active tDCS, treadmill+ sham tDCS) 2 mA, 20 min each session	No significant changes were found before and after under the tDCS+ treadmill condition.
Manenti et al. (2016)	tDCS+ physical training	20 PD-MCI	According to EGG 10–20 system **anode:** F3/F4 (contralateral to the most affected body side) **cathode:** FP2/FP1 (contralateral to the anode)	Personalized unilateral DLPFC	Placebo-controlled, double blind, randomized 2 mA tDCS per day 5 days per week 2 weeks	tDCS+ physical therapy: PD-CRS frontal-subcortical scale↑ PD-CRS total scale↑ and verbal fluency↑ PD-CRS frontal-subcortical scale and verbal fluency showed a long lasting effect of tDCS even 3 months after stimulation.
Del Felice et al. (2019)	tACS+ physical training	15 PD and 21 healthy subjects	**Stimulation electrode** : brain region where power spectral difference detected between patient and healthy controls, including FC1,FC5,C3,C4,F3,CP5 and PZ. **reference electrode:** ipsilateral mastoid	Personalized	Placebo-controlled, double blind, randomized, crossover (active tACS, sham) 1 mA minimum and 2 mA maximum in sinusoidal current relative β excess: 4 Hz; relative θ excess: 30 Hz, 30 min per day, 5 days per week, 2 weeks one session, with an 8 weeks separation between sessions.	Theta tACS stimulation showed bradykinesia item score↓ MOCA↑ only theta stimulation at right sensorimotor area and left frontal have additional neurophysiological changes: EEG frequencies↓
Biundo et al. (2015)	tDCS+ computer training	24 PD-MCI	**Anode** :individual left DLPFC **cathode**: contralateral supraorbital region.	**Left DLPFC**	Placebo-controlled, double blind, crossover (active tDCS/sham tDCS) 20 min active/sham+10 min CT per day, 4 days per week, 4 weeks	**After 4 weeks of stimulation**: attention ↓ execution functions ↓ **at week 16**: story learning test ↑ immediate memory index↑
Lawrence et al. (2018)	tDCS+ computer training	38 PD-MCI	According to EEG 10/20 system region **anode:** F3 **cathode:** FP2	**Left DLPFC**	Controlled, double blind, randomized 1.5 mA, once a week, 4 weeks, 20 min per session.	**Standard Computer training+ tDCS:** executive function↑ attention/working memory↑ memory↑ language↑ activities of daily living↑ **tailored computer training+ tDCS**: executive function↑ memory↑ language↑ **tDCS:** attention/working memory↑ memory↑
**Closed loop tES**						
Brittain et al. (2013)	tACS	14 tremor-dominant Parkinson’s disease	**Stimulation electrode**: hotspot of contralateral motor region for the most pronounced tremor muscles in the most affected upper limb **reference electrode**: shoulder contralateral to the stimulation electrode	M1	Placebo-controlled, double blind, crossover (tACS with nearest tremor frequency, tACS with double tremor frequency, sham) 10 min, 2 mA	Tremor amplitude depends on the phase alignment between tACS and tremor signals Individualized closed loop tACS: 42% ↓ in rest tremor.

*↑, increase; ↓, decrease.*

*M1: primary motor cortex; DLPFC: dorsal lateral prefrontal cortex; PFC: prefrontal cortex; SMA: supplementary motor area; TUG: Timed Up & Go test; FOG: freezing of gait; PD-CRS: The Parkinson’s Disease-Cognitive Rating Scale; HHB: hemoglobin.*

## Novel Stimulation Modes

Conventional tDCS usually target at one specific brain region, thereby excite or inhibit the activity within that region. The most frequently targeted regions for tES in PD studies are summarized in Table 2. However, to enable the ability to modulate altered brain circuit or functional network found in PD patients, the invention of novel stimulation modes is required.

**TABLE 2:** Frequently targeted brain regions for tES in Parkinson’s disease.

Brain cortex	Position in 10–20 EEG system	Reasons for targeting
Primary motor cortex (M1) (Boggio et al., 2006; Fregni et al., 2006; Dagan et al., 2018)	C3(left), C4(right), CZ	Motor: planning and execution of movement, dysfunction of M1 was found in PD (Ridding et al., 1995)
Bilateral dorsal lateral prefrontal cortex (DLPFC) (Pereira et al., 2013)	F4 (right), F3(left)	Cognitive: execution function, impaired function of rDLPFC was found in PD (Li et al., 2016; Cai et al., 2021)
Supplementary motor area (sma) (Costa-Ribeiro et al., 2017; Kami et al., 2018; Sadler et al., 2021)	FCz	Motor: planning and execution of movement, connected to diverse cortical or subcortical brain regions (Liotti et al., 2003; Mure et al., 2012)
Pre supplementary motor are(pre-SMA) (Lohse et al., 2020)	Fz	Cognitive: execution function, connected to diverse cortical and subcortical regions (Gan et al., 2020; Nakamura et al., 2001)
Cerebellum (Workman et al., 2020)	Approximately in O1, O2, Oz	Sensorimotor and cognitive function, abnormal activity in PD (Rascol et al., 1997; Yu et al., 2007)
Sensorimotor are(SM) (Ishikuro et al., 2018; Del Felice et al., 2019)	CZ,T7, C3, P7 and P3 T8, C4, P8 and P4	Both cognitive and motor: integration of sensory and motor, controls movement. Impaired function in PD (Bagnato et al., 2006)

### Multielectrode Transcranial Electrical Stimulation

Multielectrode tES is an improved version of tES with shared mechanism but multielectrode montages. Currently, multielectrode tES consists of multitarget tES and high-definition tES (hd-tES). Multitarget can modulate multiple regions of interest (Fischer et al., 2017), while hd-tES aims to achieve higher focality by minimizing the electrodes size and multiplying return electrodes (Datta et al., 2009). These two techniques are usually incorporated as a combination in experiments. In PD studies, up to date, only multitarget tDCS and hd-tDCS have been tested in clinical population.

Freezing of gait (FOG) is a characteristic symptom in PD patients and is usually seen as a target disability in PD tES studies. Stemming from both cognitive (failed at decision making) and motor impairments (failed at action) (Nutt et al., 2011), early FOG tDCS studies either targeted on M1 or left dorsolateral prefrontal cortex (lDLPFC) (Rektorova et al., 2007), with M1 stimulation aiming to alleviate FOG through motor improvements, while lDLPFC stimulation used against cognitive defects. Since both stimulation montages seemingly harvested promising results, Dagan et al. assumed that an optimized tES paradigm for FOG should improve motor and cognition at the same time. Using hd multitarget tDCS, both M1 and lDLPFC were selected. In this double-blind crossover trial, each PD FOG patient had undergone all three conditions: single target (M1) stimulation, dual-target stimulation, and sham. Subjects received 20 min of stimulation for each session with an average electric field strength of 0.25 V/m in the targeted area(s). hd-tDCS targeted at either the DLPFC or M1 was proven to be non-effective, while multitarget hd-tDCS exhibited a significantly improved gait speed and accuracy of the Stroop test (Dagan et al., 2018).

However, skepticism still exists in the assumption that multitarget tDCS would improve FOG performance, as a subsequent clinical trial with a subject population of 70 PD patients revealed the opposite results. After 2 weeks of intense treatment containing 10 stimulation sessions and 5 weeks of mild treatment with 5 stimulation sessions, despite some secondary performance improvements, no significant differences in FOG provoking test and FOG severity score were found (Manor et al., 2021).

In addition, the abovementioned studies did not assess the changes in functional connectivity. It is now generally accepted that the brain consists of many functionally connected networks. The communications and interplays among brain regions are fundamental for complex behaviors (Van Den Heuvel and Pol, 2010). Multitarget hd-tDCS provide the possibility to modulate brain networks, and has proven to be effective in modulating communications between brain regions (Yaqub et al., 2018). PD patients posed reduced functional connectivity in multiple brain networks, such as the default mode network and sensorimotor network (Ruan et al., 2020), therefore, further exploration and modulation of functional networks using multitarget hd-tDCS prove to be an efficient solution.

### Transcranial Alternating Current Stimulation

Transcranial alternating current stimulation provides a sinusoidal external current stimulation that could either synchronize or desynchronize with the internal cortical rhythm (Antal and Paulus, 2013). Dissimilar to tDCS, tACS has more viable parameters, such as amplitude, frequency, and phase. Hence, tACS is capable of specifically correcting natural brain oscillations back to normal.

Parkinson’s disease patients suffer from pathological changes in internal brain oscillation frequency ranges. Higher theta (4–8 Hz), delta (less than 4 Hz) activities and lower alpha (8–12 Hz), beta (12–30 Hz) activities in the basal ganglia were detected in PD patients (Soikkeli et al., 1991). Additionally, PD patients also exhibit a reduced power of gamma band (25–140 Hz) oscillation (Crone et al., 1998) as well as deviated task-specific neuronal oscillation activity (Possti et al., 2021).

One of the earliest experiments applying tACS toward PD used 77.5 Hz 15 mA current, with the stimulation electrode placed over the forehead and references at mastoids. The total off-medication Unified Parkinson’s Disease motor rating scale (UPDRS) of this experiment exhibited no significant difference in scores between early PD patients and sham patients (Shill et al., 2011). Nevertheless, later tACS PD studies have applied more reasonable parameters and successfully improved subjects’ behavior. Del Felice et al. constructed a study that considered the baseline neuron activity that fluctuated in different individuals. By comparing PD patients with healthy controls, PD subjects were divided into two experimental groups. If PD subjects had higher relative power than healthy subjects in the range of fast frequencies (i.e., beta waves), tACS was set at 4 Hz. If they had excessive slow frequencies (i.e., theta wave), the tACS frequency was set in the fast frequency range at 30 Hz. Theta stimulation resulted in bradykinesia item improvement in the UPDRS, and cognitive improvements assessed by the Montreal Cognitive Assessment scale (MOCA). Only theta stimulation on sensorimotor area resulted in both behavior improvements and sustained beta rhythm reduction (Del Felice et al., 2019).

Additionally, tACS can be applied to probe and detect the causal relationship between behavior and neuron activity. In a randomized crossover tACS study, the associations between bradykinesia and abnormally altered beta/gamma oscillations in primary motor cortex (M1) were exposed when 1 mA tACS at 20 Hz or 7 Hz was delivered to patients. The stimulation electrode was centered over the first dorsal interosseous hotspot, while the return electrode was centered over the PZ according to the EEG 10–20 system. As a result, the increased beta power led to aggravated bradykinesia, while gamma synchronization entrained by tACS mitigated these symptoms (Guerra et al., 2021).

To clarify how this aberrantly increased beta power is linked with bradykinesia, Krause et al. hypothesized that an increase in beta power contributed to decreased cortico-muscular coupling (CMC); a critical linkage between neurological activity and motor performance that subsequently leads to bradykinesia and akinesia. Ten PD patients and 10 healthy control subjects were recruited for this study. Each participant received three sessions of 1 mA current stimulation targeted at M1, randomly applying 15 min of 10 Hz tACS, 20 Hz tACS, and sham. To determine the effects of each stimulus, the behavior performance of PD was assessed after each session. Only after 20 Hz stimulation did PD patients reveal a more rigid motor performance and a significant reduction in beta band CMC amplitude, while healthy controls experienced no significant difference in CMC and behavioral performance after either 10 or 20 Hz stimulation (Krause et al., 2013).

Despite these positive results, tACS might induce phosphene, namely, the feeling of flickering lights. Phosphene could affect subjects’ performance during tasks and impair the reliability of double-blind studies. Even if the phosphene threshold could be increased to 80 Hz when targeting brain regions far from the retina, it was found that maximum phosphene levels could occur between 14 and 20 Hz under an occiput–vertex electrode montage (Schutter and Hortensius, 2010). Thus, solutions are required for those phosphene inducing montages.

### Transcranial Pulsed Current Stimulation

Different from conventional tDCS, tPCS provides discontinuous direct current interrupted by either short or long periodical inter-pulse intervals (Ma et al., 2019), thus adding two additional parameters: inter-pulse intervals and pulse durations. Although phosphene might also be induced by tPCS, the overall tolerability toward tPCS is better than tDCS in healthy subjects, with a significantly reduced feeling of itching, tingling, and eye flashing (Jaberzadeh et al., 2014).

In a Parkinsonian study, a combined tPCS and treadmill strategy was applied for 10 PD freezing of gait(FOG) patients, with both pulse duration and inter-pulse interval of tPCS being 33.3 μs. The tPCS targeted M1 provided 20 min of stimulation with or without concurrent treadmill training. The gait speed and gait amplitude, however, were found to be improved after a single tPCS session but not in the combined group or treadmill alone group (Alon et al., 2012). However, this study did not include sham tPCS as a placebo control, and the reliability of its results was therefore dented. Further studies should take place to further assess the efficacy of tPCS toward PD.

There was a study toward healthy population implicated that the duration of tPCS was not aligned with the lasting time of aftereffects (Vasquez et al., 2016). Thus, the non-linear effects of tPCS together with its unclear mechanisms complicate the process of selecting proper tPCS parameters. Consequently, few PD studies have focused on tPCS.

### Transcranial Random Noise Stimulation

Transcranial random noise stimulation delivers alternating currents of random frequencies and amplitudes within a specific range of the spectrum (Carvalho et al., 2018). tRNS has been used as an active control in several neuromodulation studies; nevertheless, tRNS itself can also serve as a possible therapeutic stimulation method for PD. Like tACS, tRNS can also interfere with internal brain oscillations and neuronal activities. In healthy controls, weak tRNS over M1 increased corticospinal excitability during and after stimulation (Terney et al., 2008). In addition, tRNS exhibits a higher perception threshold (Ambrus et al., 2010) and is more effective activating M1 (Moliadze et al., 2014) than anodal tDCS in healthy controls.

In order to assess the potential of tRNS toward PD, a study has combined tRNS with intermittent theta burst stimulation (iTBS) to detect neuron activity alterations. In this study, PD patients who received a maximum 640 Hz 0.5 mA tRNS over M1 showed an adverse effect on the excitability of M1. In contrast, in healthy controls, M1 cortical excitability was enhanced after stimulation, while PD patients exhibited decreased cortical activity (Stephani et al., 2011). This study revealed a difference in tRNS efficacy among different populations. Thus, further assessment of the efficacy of tRNS as a rehabilitation strategy for PD is needed.

In a double-blind study specific to PD with major cognitive impairment (PD-MCI), Monastero et al. applied 1.5 mA tRNS oscillating randomly between 100 and 600 Hz on M1 to 10 PD-MCI subjects. In this study, 10 patients went through two randomized stimulation sessions, one of them being an active tRNS session and the other being sham. As a result, the total UPDRS score of subjects was significantly increased only after the active tRNS session (Monastero et al., 2020). Again, the validity of this result is doubtful due to the limited subject population and the lack of repetitive sessions.

### Outlook for Novel Stimulation Modes

Although promising results were shown by these novel approaches of tES, some studies revealed adverse results. For example, identical multitarget hd-tDCS stimuli resulted in controversial responses in two studies, possibly attributed to the different scales of subjects. In the future, numerous studies with larger sample sizes are needed to prove the prevalence of their efficacy toward PD. In addition, due to the substantial variabilities between populations, even if studies have revealed positive results toward healthy subjects or other homologous disorders, the stimulation parameters should be carefully reconsidered when applied to PD patients.

The investigation of tES mechanisms can not only provide insight into finding optimized tES parameters but also help to explore the neurological process behind specific symptoms. Many studies have employed novel tES to investigate the causal relationship between specific oscillation patterns and behaviors. The understanding of behavior mechanisms can in turn help the design of effective interventions, forming a virtuous circle.

Conventional tDCS separates the whole brain into individual functional regions and neglects the brain’s functional networks, while novel modes of tES provide the possibility to modulate the communications between regions. Future studies should focus on stimulating the functional connectivity within brain networks.

Although the mechanisms behind tRNS and tPCS are yet uncertain, future exploration of their mechanisms might be extremely advantageous to optimize parameter settings, that will encourage more tRNS and tPCS studies. Therefore, the adjustment of stimulation would be quite challenging and might lead to contradictory results.

In addition, tPCS and tACS both face phosphene problems. Although there are solutions such as masking flicker stimuli to offset the phosphene (Krause et al., 2013), new methods that eliminate phosphene generation are likely better alternatives.

Recently, the tolerability and feasibility of remotely supervised tDCS (rs-tDCS) has been validated in PD patients, and promising therapeutic improvements were shown by rs-tDCS targeted at the DLPFC paired with cognitive training (Dobbs et al., 2018). This remotely supervised tES provides hints for future studies.

Single session tES usually experienced shorter aftereffects, while repetitive sessions of stimulation would harvest longer aftereffects duration. A single session tDCS or tACS (Neuling et al., 2013; Wach et al., 2013) usually leads to aftereffects that last 30 min or so, independent of the stimulation time. However, a study revealed that repetitive sessions of tDCS with short intervals(several minutes) can harvest up to 24 h of offline effects, while longer intervals(several hours) was found with little or no effects on plasticity (Agboada et al., 2020). Future studies are required to validate whether the short interval repetitive tES would results in sustained and wide-spread aftereffects.

## Transcranial Electrical Stimulation Combined With Other Intervention

In addition to the novel stimulation approaches discussed above, the combination of existing tES with another PD therapy can also be recognized as a novel stimulation method.

### Transcranial Electrical Stimulation Combined With Drug

Since one limitation of pharmacologic therapy is side effects, it is intriguing to discuss whether the combination of tES and drugs would optimize improvements and lessen side effects at the same time.

In contrast to tDCS experiments where medication is controlled as baseline, in the study of Ferrucci et al., tDCS was provided as a supplementary intervention of levodopa to eliminate L-dopa-induced cognitive and motor impairments. M1 tDCS, cerebellar tDCS, and sham sessions are randomized for each subject, and each session was at least 1 month separated from another. Anodal tDCS (2 mA) was applied for 20 min in 5 consecutive days in each active stimulation session. Although no other cognitive or motor improvements were observed, both M1 and cerebellar tDCS led to a decrease in levodopa-induced dyskinesias in PD patients (Ferrucci et al., 2016).

### Transcranial Electrical Stimulation Combined With Transcranial Magnetic Stimulation

Transcranial electrical stimulation can also be combine with TMS to boost the benefits of intervention. Recently, a dual mode tDCS scheme applied anodal tDCS together with repetitive transcranial magnetic stimulation (rTMS). The stimulation electrode of tDCS was placed over the lDLPFC, while TMS targeted M1. This stimulation scheme demonstrated a significant improvement in the executive functions of PD patients under dual-mode stimulation conditions (Chang et al., 2017).

Similarly, tACS delivered at gamma frequency combined with intermittent theta burst stimulation (iTBS) can also repair the damaged brain activity of PD patients. iTBS is a novel rTMS that provides more tolerable and robust action than conventional rTMS (Sanna et al., 2019). In PD patients, the long-term potentiation (LTP)-like plasticity induced by iTBS is impaired in M1 which subsequently damages PD patients’ motor learning ability (Ziemann et al., 2006). Concurrently, PD patients also present decreased gamma oscillatory activity in the basal ganglia-thalamo-cortical network. By applying 70 Hz tACS together with iTBS, iTBS-induced LTP-like plasticity in PD patients can be recovered (Guerra et al., 2020). Consequently, behavior improvements of this repairment could be assessed in future studies.

### Transcranial Electrical Stimulation Combined With Physical or Cognitive Training

Motor and cognitive training can effectively ameliorate PD symptoms (Sidaway et al., 2006; Leung et al., 2015). Hence it might be possible to harvest the benefits of training and tES by combining these interventions.

In PD-MCI patients, tDCS combined with physical training showed steady cognitive improvements, but no significant additional effects of tDCS was found (Manenti et al., 2016). In line with this finding, Fernández-Lago et al. found that after a single 20 min session of active tDCS stimulation with treadmill walking training, there was no enhancement of tDCS toward sole treadmill walking training (Fernandez-Lago et al., 2017).

In contrast, in another sham-controlled, double-blind crossover study, Kaski et al. applied tDCS at M1 and the premotor cortex, and together with physical training, PD patients were found to have improved gait and balance performance (Kaski et al., 2014). Another study that combined 30 min of aerobic exercise with anodal tDCS targeted at the prefrontal cortex (PFC) revealed a positive impact of this combination on PFC activity, gait, and cognition (Conceição et al., 2021).

For cognitive training, another study combined computer-based cognitive training (CT) with 2 mA tDCS targeted at the DLPFC. This combined intervention was administered 4 times a week for 4 weeks, and at the week 16 follow-up survey, the impairment in executive skill and attention in PD-MCI patients was ameliorated and the immediate memory skill ability increased (Biundo et al., 2015). A later study divided computer-based CT into standard and tailored CT. Standard CT was more general, while tailored CT was specific to types of impairments, but both CT together with tDCS over the lDLPFC at 2 mA, harvested improvements in PD patients’ cognitive behavior, such as attention and working memory (Lawrence et al., 2018).

### Outlook for Transcranial Electrical Stimulation Combined With Other Interventions

Since different interventions act through distinct mechanisms to alleviate PD symptoms, combined interventions can modulate multiple pathways with higher efficiency. Future studies might be able to provide individualized combination schemes according to patients’ symptoms and pathogenic factors, leading to maximized improvements and minimized side-effects in individuals.

Nevertheless, some limitations in combined interventions need to be considered.

First, TMS itself has already faced the problems of tolerability and unblinding toward patients and adding on TMS might worsen the situation.

In addition, although many have applied combination interventions for PD, few of them include control groups to assess the improvement of dual therapy interventions compared to single intervention. Therefore, in some studies, it is uncertain whether these combinations are better, more studies and a larger number of subjects are required for further validation of their effects.

Moreover, determining the framework of combined intervention is also challenging, since several combined stimulation studies have focused on the yet elusive mechanisms. The combination of interventions might support or interact with each other, forming complex response networks. Therefore, it is difficult to decide the dose and timing of interventions. Should these interventions be given together, or which takes precedence? How can the dose be decided to optimize the efficacy while at the same time not resulting in unpleasant feelings or even damage? These questions need to be solved for a better understanding toward PD.

## Closed Loop Transcranial Electrical Stimulation

It is generally accepted that discrepancies in effects can emerge between individuals when receiving identical stimulation. Similarly, several studies have witnessed deviations in neuronal feedback between different circumstances undergone by the same subject. To date, most tES research on PD has focused on open-loop schemes, providing constant predefined stimulation during the whole experimental process. However, due to these irremovable inter- and intra-subject variabilities, side effects, and low effect sizes may occur during clinical applications (Iturrate et al., 2018). Therefore, closed loop tES, which is self-adaptive toward changes in brain activity, Therefore, closed loop tES has become a hot topic.

Closed loop tES can provide self-adaptive stimulation according to the feedback of brain activity, forming a feedback-control close loop between input and output signals. To be more specific, when a predefined signal is detected(could be both behavior or brain activity) by the sensor, closed loop system should immediately receive the feedback from the sensor and select the corresponding stimulation parameters, then pass to the stimulator to provide real-time control.

Brittain et al. explored a closed loop tACS strategy toward rest tremor, a dominant motor symptom of PD unresolved by dopaminergic therapy. First, the patient-specific tremor frequencies were recorded, then tACS at tremor frequency was applied to M1 at original or double tremor frequencies. The amplitude of tremor was found to be minimized when the tremor signal and tACS were in phase and when stimulation was set at tremor frequency. Then, subjects were stimulated for 30 s at individual optimal tACS phases with arbitrary input. By alignment of internal and external oscillations, the phase of this pathogenic cortical oscillation would be canceled out, hence inhibiting an average of 42% of resting tremors (Brittain et al., 2013). This closed-loop tremor suppression tACS system might provide a basis for future Parkinsonian studies.

Ideally, optimized closed-loop tES should be able to recognize diverse circumstances and select symptom-specific tES parameters, in which case the specificity of stimulation would be improved, like methodology discussed above against rest tremor.

Additionally, an ideal closed loop tES should also automatically switch on when symptoms evoked and switch off when symptoms disappear, reducing the effect of overtreatment, and the financial cost of stimulation. For instance, A research group have designed a closed-loop EEG-tDCS system. They predefined an EEG threshold for automatic stimulation initiation, which in this study was when subjects were performing Stroop test. After the stimulation session, the sensor would again search for any above-threshold event and prepared to initiate another tDCS session. This closed loop EEG-tDCS system had achieved this system achieved 100% correct activation of tDCS in healthy population (Leite et al., 2017).

A recent DBS study on primates verified improved efficiency of closed-loop methodology. By using an adaptive GPi-DBS scheme that responds to signal changes in M1, the improvements in parkinsonian symptoms were greater than normalized DBS at a lower firing rate of GPi (Rosin et al., 2011). There are now closed-loop DBS that provide in-time stimulation corresponding to neuronal signals, harvesting the same therapeutic effects with less energy consumption (Swann et al., 2018).

Current studies applying closed-loop schemes toward PD are mostly focused on DBS, given the preliminary encouraging results, however, conducting a closed-loop tES scheme seems a very promising field in neuronal modulation of PD. Although few closed-loop tES studies focused on PD treatment, the applicability and efficacy of closed- loop tES has been validated in epilepsy (Berenyi et al., 2012; Kozak and Berenyi, 2017) and sleep (Ketz et al., 2018; Cellini et al., 2019; Hubbard et al., 2021).

There are two possible strategies in conducting closed loop tES studies. First, like DBS, tES might be able to track the internal oscillation of the brain, and if any abnormal oscillations were detected, tES would be activated and initiated the stimulation. On the other hand, closed loop tES can monitor the behavior of subjects and apply stimulation corresponding to subjects’ performance. For example, in the above study of resting tremors, the amplitude and frequency of tremors were recorded to deliver specific stimulation. These external behavior data would serve as an indicator for stimulation settings. Recently, a study assessed the capability of a smartwatch to track the time when PD patients suffer severe resting tremor outbreaks. In the future, this kind of device might be adopted in a closed-loop PD intervention study, providing precise behavioral data (Powers et al., 2021).

Although no other tES closed-loop study has focused on PD, researchers are trying to investigate novel tES equipment that can satisfy the demands of closed-loop stimulation. For example, a brain-machine interface system that integrate frequency-domain near infrared spectroscopy (fdNIRS) input and tDCS output was designed to enable the application of closed loop tDCS (Miao and Koomson, 2018). In addition, a recently developed tool box called brain electrophysiological recording and stimulation(BEST), had expanded the range of input recording and output stimulation devices with improved compatibility (Hassan et al., 2022). These systems and algorithms would enable the real-time feedback control of input signals by providing a platform for input and output signal integrating and processing, providing technological pilar for future closed-loop studies.

### Outlook for Closed Loop Transcranial Electrical Stimulation

Although closed-loop tES studies toward PD are few, adaptive stimulation schemes have gained vast attention since they can deliver individual- or circumstance-specific modulation. Self-adaptive individualized closed-loop scheme can achieve higher efficacy; and closed-loop tES schemes in the future might also be able to automatically switch on when symptoms occur, like closed-loop DBS.

Another serious technical problem for closed loop tES is the time delay between detection of changes in brain conditions and the response to stimulus. For symptoms with a short provoking duration, the time delay in response might just miss the period of symptoms and become ineffective. Future studies might employ machine learning to predict the occurrence of symptoms so that more precise stimulation could be provided. In addition, using a brain machine interface (BMI) can provide a quicker stimulation response. For example, in a chronic pain study that used BMI in mice to monitor and stimulate the brain, the time delay between characteristics detected and stimulation response was minimized; thus, the pain could be released even when recognizing acute pain signals (Zhang et al., 2021).

Many studies have proposed numerous novel adaptive tACS and tDCS systems that proved to be safe and tolerable in healthy people (Leite et al., 2017; Guo et al., 2019). Building on these results, investigators could adopt those technologies in future closed loop tES studies in clinical PD populations.

## Conclusion

Novel tES has proven their potential in preventing both motor and cognitive symptoms in PD and has been clinically translated as a supplementary intervention.

Overall, these novel patterns seek to achieve better performance and safety based on achievements made by conventional tES. These three novel tES methods is complementary with each other in a progressive manner. Novel tES approaches provide the possibility to stimulate the brain as functional networks instead of individual regions and have been proved to be effective in most of the PD studies, enhancing current understanding of PD pathology. The development of novel tES modes expanded the choice for combining tES with other intervention strategies and promote the possibility in obtaining the ideal multi-pathway-targeted combination, achieving a “1 + 1 > 2” improvement in efficacy as well as a reduced side-effect. Furthermore, considered as the most promising tES strategy, closed loop tES could take individual differences into consideration and adapt to the patient’s response, further optimizing therapeutic specificity. Nevertheless, closed-loop tES requires knowledge of pathological mechanisms and optimized stimulation pattern for each symptom, thus help the recognition of symptom-related activity and matching corresponding stimulation for recognized symptom.

Nevertheless, the results between some of the novel tES studies remain inconsistent, which might be resulted from the small sample size in most of the studies. To put novel tES into clinical use, more clinical trials with larger participant numbers are required. In addition, future studies should recognize the brain as a cohort of many functional networks and utilize tES to modulate functional connectivity between functional regions during PD symptoms. Apart from the study design, there should be more studies that investigate closed loop tES, providing higher specificity of stimulation with the lower consumption of energy. Moreover, efforts should be devoted to the invention of novel tES devices with higher operability that can provide in-home stimulation, making this therapy more convenient and available for patients, for example, rs-tES.

Currently, tES strategies lead to undesirable sensations of stimulation that hinder wider applications. Many tES studies are now focusing on temporal interference stimulation, which seems to be almost imperceptible to healthy subjects (Ma et al., 2021). Future studies might adopt this strategy to avoid unblinding of patients in double-blind studies, providing more pleasant stimulation experience for patients.

In conclusion, novel tES patterns have harvested encouraging improvements in both motor and cognitive symptoms of Parkinson’s disease despite flaws.

## Author Contributions

JB, RN, LY, YZu, YY, and QM designed the study. RN compiled and analyzed the data and wrote the manuscript. JB and RN were closely involved in the definition of the manuscript structure. JB, ZC, and YZo provided critical feedback on all the tables and text. JB, WY, and RN provided support for the methodical work and supervision. All authors read and approved the final manuscript.

## Conflict of Interest

The authors declare that the research was conducted in the absence of any commercial or financial relationships that could be construed as a potential conflict of interest.

## Publisher’s Note

All claims expressed in this article are solely those of the authors and do not necessarily represent those of their affiliated organizations, or those of the publisher, the editors and the reviewers. Any product that may be evaluated in this article, or claim that may be made by its manufacturer, is not guaranteed or endorsed by the publisher.

## References

[B1] Agboada D. Mosayebi-Samani M. Kuo M. F. Nitsche M. A. (2020). Induction of long-term potentiation-like plasticity in the primary motor cortex with repeated anodal transcranial direct current stimulation - Better effects with intensified protocols? *Brain Stimul.* 13 987–997. 10.1016/j.brs.2020.04.009 32325264

[B2] Alon G. Yungher D. A. Shulman L. M. Rogers M. W. (2012). Safety and immediate effect of noninvasive transcranial pulsed current stimulation on gait and balance in Parkinson disease. *Neurorehabil. Neural. Repair.* 26 1089–1095. 10.1177/1545968312448233 22581566

[B3] Ambrus G. G. Paulus W. Antal A. (2010). Cutaneous perception thresholds of electrical stimulation methods: comparison of tDCS and tRNS. *Clin. Neurophysiol.* 121 1908–1914. 10.1016/j.clinph.2010.04.020 20471313

[B4] Antal A. Paulus W. (2013). Transcranial alternating current stimulation (tACS). *Front. Hum. Neurosci.* 7:317. 10.3389/fnhum.2013.00317 23825454 PMC3695369

[B5] Bagnato S. Agostino R. Modugno N. Quartarone A. Berardelli A. (2006). Plasticity of the motor cortex in Parkinson’s disease patients on and off therapy. *Mov. Disord.* 21 639–645. 10.1002/mds.20778 16353175

[B6] Beitz J. M. (2014). Parkinson’s disease: a review. *Front. Biosci.* 6 65–74. 10.2741/s415 24389262

[B7] Berenyi A. Belluscio M. Mao D. Buzsaki G. (2012). Closed-loop control of epilepsy by transcranial electrical stimulation. *Science* 337 735–737. 10.1126/science.1223154 22879515 PMC4908579

[B8] Biundo R. Weis L. Fiorenzato E. Gentile G. Giglio M. Schifano R. (2015). Double-blind Randomized Trial of tDCS Versus Sham in Parkinson Patients With Mild Cognitive Impairment Receiving Cognitive Training. *Brain Stimul.* 8 1223–1225. 26319357 10.1016/j.brs.2015.07.043

[B9] Boggio P. S. Ferrucci R. Rigonatti S. P. Covre P. Nitsche M. Pascual-Leone A. (2006). Effects of transcranial direct current stimulation on working memory in patients with Parkinson’s disease. *J. Neurol. Sci.* 249 31–38. 10.1016/j.jns.2006.05.062 16843494

[B10] Boord P. Madhyastha T. M. Askren M. K. Grabowski T. J. (2017). Executive attention networks show altered relationship with default mode network in PD. *Neuroimage Clin.* 13 1–8. 10.1016/j.nicl.2016.11.004 27896064 PMC5121155

[B11] Brittain J. S. Probert-Smith P. Aziz T. Z. Brown P. (2013). Tremor suppression by rhythmic transcranial current stimulation. *Curr. Biol.* 23 436–440. 10.1016/j.cub.2013.01.068 23416101 PMC3629558

[B12] Bueno M. E. B. do Nascimento Neto L. I. Terra M. B. Barboza N. M. Okano A. H. Smaili S. M. (2019). Effectiveness of acute transcranial direct current stimulation on non-motor and motor symptoms in Parkinson’s disease. *Neurosci. Lett.* 696 46–51. 10.1016/j.neulet.2018.12.017 30553865

[B13] Cai M. Dang G. Su X. Zhu L. Shi X. Che S. (2021). Identifying mild cognitive impairment in Parkinson’s disease with electroencephalogram functional connectivity. *Front. Aging Neurosci.* 381:701499. 10.3389/fnagi.2021.701499 34276350 PMC8281812

[B14] Carvalho S. Leite J. Fregni F. (2018). *Transcranial alternating current stimulation and transcranial random noise stimulation. Neuromodulation.* Berlin: Elsevier, 1611–1617.

[B15] Cellini N. Shimizu R. E. Connolly P. M. Armstrong D. M. Hernandez L. T. Polakiewicz A. G. (2019). Short Duration Repetitive Transcranial Electrical Stimulation During Sleep Enhances Declarative Memory of Facts. *Front. Hum. Neurosci.* 13:123. 10.3389/fnhum.2019.00123 31031612 PMC6474382

[B16] Chang W. H. Kim M. S. Park E. Cho J. W. Youn J. Kim Y. K. (2017). Effect of Dual-Mode and Dual-Site Noninvasive Brain Stimulation on Freezing of Gait in Patients With Parkinson Disease. *Arch. Phys. Med. Rehabil.* 98 1283–1290. 10.1016/j.apmr.2017.01.011 28193533

[B17] Conceição N. R. Gobbi L. T. B. Nobrega-Sousa P. Orcioli-Silva D. Beretta V. S. Lirani-Silva E. (2021). Aerobic Exercise Combined With Transcranial Direct Current Stimulation Over the Prefrontal Cortex in Parkinson Disease: Effects on Cortical Activity. Gait, and Cognition. *Neurorehabil. Neural. Repair.* 35 717–728. 10.1177/15459683211019344 34047235

[B18] Costa-Ribeiro A. Maux A. Bosford T. Aoki Y. Castro R. Baltar A. (2017). Transcranial direct current stimulation associated with gait training in Parkinson’s disease: a pilot randomized clinical trial. *Dev. Neurorehabil.* 20 121–128. 10.3109/17518423.2015 26864140

[B19] Crone N. E. Miglioretti D. L. Gordon B. Lesser R. P. (1998). Functional mapping of human sensorimotor cortex with electrocorticographic spectral analysis. II. Event-related synchronization in the gamma band. *Brain* 121 2301–2315. 10.1093/brain/121.12.2301 9874481

[B20] Dagan M. Herman T. Harrison R. Zhou J. Giladi N. Ruffini G. (2018). Multitarget transcranial direct current stimulation for freezing of gait in Parkinson’s disease. *Mov. Disord.* 33 642–646. 10.1002/mds.27300 29436740 PMC5964604

[B21] Datta A. Bansal V. Diaz J. Patel J. Reato D. Bikson M. (2009). Gyri-precise head model of transcranial direct current stimulation: improved spatial focality using a ring electrode versus conventional rectangular pad. *Brain Stimul.* 2 201–7,7e1. 10.1016/j.brs.2009.03.005 20648973 PMC2790295

[B22] Del Felice A. Castiglia L. Formaggio E. Cattelan M. Scarpa B. Manganotti P. (2019). Personalized transcranial alternating current stimulation (tACS) and physical therapy to treat motor and cognitive symptoms in Parkinson’s disease: a randomized cross-over trial. *NeuroImage.* 22:101768. 10.1016/j.nicl.2019.101768 30921609 PMC6439208

[B23] Dobbs B. Pawlak N. Biagioni M. Agarwal S. Shaw M. Pilloni G. (2018). Generalizing remotely supervised transcranial direct current stimulation (tDCS): feasibility and benefit in Parkinson’s disease. *J. Neuroeng. Rehabil.* 15:114. 10.1186/s12984-018-0457-9 30522497 PMC6284269

[B24] Doruk D. Gray Z. Bravo G. L. Pascual-Leone A. Fregni F. (2014). Effects of tDCS on executive function in Parkinson’s disease. *Neurosci. Lett.* 582 27–31. 10.1016/j.neulet.2014.08.043 25179996

[B25] Fabbri M. Coelho M. Guedes L. C. Chendo I. Sousa C. Rosa M. M. (2017). Response of non-motor symptoms to levodopa in late-stage Parkinson’s disease: Results of a levodopa challenge test. *Parkinsonism Relat. Dis.* 39 37–43. 10.1016/j.parkreldis.2017.02.007 28389156

[B26] Fernandez-Lago H. Bello O. Mora-Cerda F. Montero-Camara J. Fernandez-Del-Olmo M. A. (2017). Treadmill Walking Combined With Anodal Transcranial Direct Current Stimulation in Parkinson Disease: A Pilot Study of Kinematic and Neurophysiological Effects. *Am. J. Phys. Med. Rehabil.* 96 801–808. 10.1097/PHM.0000000000000751 28398968

[B27] Ferrucci R. Cortese F. Bianchi M. Pittera D. Turrone R. Bocci T. (2016). Cerebellar and Motor Cortical Transcranial Stimulation Decrease Levodopa-Induced Dyskinesias in Parkinson’s Disease. *Cerebellum* 15 43–47. 10.1007/s12311-015-0737-x 26542731

[B28] Fischer D. B. Fried P. J. Ruffini G. Ripolles O. Salvador R. Banus J. (2017). Multifocal tDCS targeting the resting state motor network increases cortical excitability beyond traditional tDCS targeting unilateral motor cortex. *Neuroimage* 157 34–44. 10.1016/j.neuroimage.2017.05.060 28572060 PMC12824506

[B29] Fregni F. Boggio P. S. Santos M. C. Lima M. Vieira A. L. Rigonatti S. P. (2006). Noninvasive cortical stimulation with transcranial direct current stimulation in Parkinson’s disease. *Mov. Disord.* 21 1693–1702. 10.1002/mds.21012 16817194

[B30] Gan C. Wang M. Si Q. Yuan Y. Zhi Y. Wang L. (2020). Altered interhemispheric synchrony in Parkinson’s disease patients with levodopa-induced dyskinesias. *NPJ Parkinsons Dis.* 6 1–7. 10.1038/s41531-020-0116-2 32665973 PMC7343784

[B31] Ganguly J. Murgai A. Sharma S. Aur D. Jog M. (2020). Non-invasive Transcranial Electrical Stimulation in Movement Disorders. *Front. Neurosci.* 14:522. 10.3389/fnins.2020.00522 32581682 PMC7290124

[B32] Guerra A. Asci F. D’Onofrio V. Sveva V. Bologna M. Fabbrini G. (2020). Enhancing Gamma Oscillations Restores Primary Motor Cortex Plasticity in Parkinson’s Disease. *J. Neurosci.* 40 4788–4796. 10.1523/JNEUROSCI.0357-20.2020 32430296 PMC7294804

[B33] Guerra A. Colella D. Giangrosso M. Cannavacciuolo A. Paparella G. Fabbrini G. (2021). Driving motor cortex oscillations modulates bradykinesia in Parkinson’s disease. *Brain* 10:awab257. 10.1093/brain/awab257 34245244

[B34] Guo D. yan Li H. Sun M. (eds) (2019). *Adaptive alternating current transcranial electrical stimulation (tACS). 2019 6th International Conference on Systems and Informatics (ICSAI).* Piscataway: IEEE

[B35] Hassan U. Pillen S. Zrenner C. Bergmann T. O. (2022). The Brain Electrophysiological recording & STimulation (BEST) toolbox. *Brain Stimul.* 15 109–115. 34826626 10.1016/j.brs.2021.11.017

[B36] Hubbard R. J. Zadeh I. Jones A. P. Robert B. Bryant N. B. Clark V. P. (2021). Brain connectivity alterations during sleep by closed-loop transcranial neurostimulation predict metamemory sensitivity. *Netw. Neurosci.* 5 734–756. 10.1162/netn_a_00201 34746625 PMC8567828

[B37] Ishikuro K. Dougu N. Nukui T. Yamamoto M. Nakatsuji Y. Kuroda S. (2018). Effects of transcranial direct current stimulation (tDCS) over the frontal polar area on motor and executive functions in Parkinson’s disease; a pilot study. *Front. Aging Neurosci.* 10:231. 10.3389/fnagi.2018.00231 30104971 PMC6077209

[B38] Iturrate I. Pereira M. Millán J. D. R. (2018). Closed-loop electrical neurostimulation: challenges and opportunities. *Curr. Opin. Biomed. Eng.* 8 28–37. 10.3389/fnins.2019.00936 31572109 PMC6751331

[B39] Jaberzadeh S. Bastani A. Zoghi M. (2014). Anodal transcranial pulsed current stimulation: A novel technique to enhance corticospinal excitability. *Clin. Neurophysiol.* 125 344–351. 10.1016/j.clinph.2013.08.025 24074626

[B40] Kami A. T. Sadler C. Nantel J. Carlsen A. N. (2018). Transcranial direct current stimulation (TDCS) over supplementary motor area (SMA) improves upper limb movement in individuals with Parkinson’s disease. *J. Exerc. Mov. Sport* 50:36. 10.1016/j.clinph.2021.06.031 34412968

[B41] Kaski D. Dominguez R. O. Allum J. H. Islam A. F. Bronstein A. M. (2014). Combining physical training with transcranial direct current stimulation to improve gait in Parkinson’s disease: a pilot randomized controlled study. *Clin Rehabil.* 28 1115–1124. 10.1177/0269215514534277 24849794

[B42] Ketz N. Jones A. P. Bryant N. B. Clark V. P. Pilly P. K. (2018). Closed-Loop Slow-Wave tACS Improves Sleep-Dependent Long-Term Memory Generalization by Modulating Endogenous Oscillations. *J. Neurosci.* 38 7314–7326. 10.1523/JNEUROSCI.0273-18.2018 30037830 PMC6596034

[B43] Kozak G. Berenyi A. (2017). Sustained efficacy of closed loop electrical stimulation for long-term treatment of absence epilepsy in rats. *Sci. Rep.* 7:6300. 10.1038/s41598-017-06684-0 28740261 PMC5524708

[B44] Krause V. Wach C. Sudmeyer M. Ferrea S. Schnitzler A. Pollok B. (2013). Cortico-muscular coupling and motor performance are modulated by 20 Hz transcranial alternating current stimulation (tACS) in Parkinson’s disease. *Front. Hum. Neurosci.* 7:928. 10.3389/fnhum.2013.00928 24474912 PMC3893573

[B45] Lau C. I. Liu M. N. Chang K. C. Chang A. Bai C. H. Tseng C. S. (2019). Effect of single-session transcranial direct current stimulation on cognition in Parkinson’s disease. *CNS Neurosci. Ther.* 25 1237–1243. 10.1111/cns.13210 31424182 PMC6834682

[B46] Lawrence B. J. Gasson N. Johnson A. R. Booth L. Loftus A. M. (2018). Cognitive Training and Transcranial Direct Current Stimulation for Mild Cognitive Impairment in Parkinson’s Disease: A Randomized Controlled Trial. *Park. Dis.* 2018:4318475. 10.1155/2018/4318475 29780572 PMC5892209

[B47] Leite J. Morales-Quezada L. Carvalho S. Thibaut A. Doruk D. Chen C. F. (2017). Surface EEG-Transcranial Direct Current Stimulation (tDCS) Closed-Loop System. *Int. J. Neural. Syst.* 27:1750026. 10.1142/S0129065717500265 28587498 PMC5527347

[B48] Leung I. H. Walton C. C. Hallock H. Lewis S. J. Valenzuela M. Lampit A. (2015). Cognitive training in Parkinson disease: A systematic review and meta-analysis. *Neurology.* 85 1843–1851. 26519540 10.1212/WNL.0000000000002145 PMC4662707

[B49] Li Y. Liang P. Jia X. Li K. (2016). Abnormal regional homogeneity in Parkinson’s disease: a resting state fMRI study. *Clin. Radiol.* 71 e28–e34. 10.1016/j.crad.2015.10.006 26628410

[B50] Liotti M. Ramig L. Vogel D. New P. Cook C. Ingham R. (2003). Hypophonia in Parkinson’s disease: neural correlates of voice treatment revealed by PET. *Neurology* 60 432–440. 10.1212/wnl.60.3.432 12578924

[B51] Lohse A. Meder D. Nielsen S. Lund A. E. Herz D. M. Løkkegaard A. (2020). Low-frequency transcranial stimulation of pre-supplementary motor area alleviates levodopa-induced dyskinesia in Parkinson’s disease: a randomized cross-over trial. *Brain Commun.* 2:fcaa147. 10.1093/braincomms/fcaa147 33225277 PMC7667528

[B52] Ma R. Xia X. Zhang W. Lu Z. Wu Q. Cui J. (2021). High Gamma and Beta Temporal Interference Stimulation in the Human Motor Cortex Improves Motor Functions. *bioRxiv* [Preprint]. 10.1101/2021.03.26.437107 PMC8761631 35046771

[B53] Ma Z. Du X. Wang F. Ding R. Li Y. Liu A. (2019). Cortical Plasticity Induced by Anodal Transcranial Pulsed Current Stimulation Investigated by Combining Two-Photon Imaging and Electrophysiological Recording. *Front. Cell Neurosci.* 13:400. 10.3389/fncel.2019.00400 31555097 PMC6727068

[B54] Manenti R. Brambilla M. Benussi A. Rosini S. Cobelli C. Ferrari C. (2016). Mild cognitive impairment in Parkinson’s disease is improved by transcranial direct current stimulation combined with physical therapy. *Mov. Disord.* 31 715–724. 26880536 10.1002/mds.26561

[B55] Manor B. Dagan M. Herman T. Gouskova N. A. Vanderhorst V. G. Giladi N. (2021). Multitarget Transcranial Electrical Stimulation for Freezing of Gait: A Randomized Controlled Trial. *Mov Disord.* 36 2693–2698. 10.1002/mds.28759 34406695

[B56] Miao Y. Koomson V. J. A. C. M. O. S. - (2018). Based Bidirectional Brain Machine Interface System With Integrated fdNIRS and tDCS for Closed-Loop Brain Stimulation. *IEEE Trans. Biomed. Circuits Syst.* 12 554–563. 10.1109/TBCAS.2018.2798924 29877819

[B57] Moliadze V. Fritzsche G. Antal A. (2014). Comparing the efficacy of excitatory transcranial stimulation methods measuring motor evoked potentials. *Neural. Plast.* 2014:837141. 10.1155/2014/837141 24804104 PMC3997131

[B58] Monastero R. Baschi R. Nicoletti A. Pilati L. Pagano L. Cicero C. E. (2020). Transcranial random noise stimulation over the primary motor cortex in PD-MCI patients: a crossover, randomized, sham-controlled study. *J. Neural. Transm..* 127 1589–1597. 10.1007/s00702-020-02255-2 32965593 PMC7666273

[B59] Morishita T. Hilliard J. D. Okun M. S. Neal D. Nestor K. A. Peace D. (2017). Postoperative lead migration in deep brain stimulation surgery: Incidence, risk factors, and clinical impact. *PLoS One* 12:e0183711. 10.1371/journal.pone.0183711 28902876 PMC5597118

[B60] Mure H. Tang C. C. Argyelan M. Ghilardi M.-F. Kaplitt M. G. Dhawan V. (2012). Improved sequence learning with subthalamic nucleus deep brain stimulation: evidence for treatment-specific network modulation. *J. Neurosci.* 32 2804–2813. 10.1523/JNEUROSCI.4331-11.2012. 22357863 PMC4557784

[B61] Nakamura T. Ghilardi M. Mentis M. Dhawan V. Fukuda M. Hacking A. (2001). Functional networks in motor sequence learning: abnormal topographies in Parkinson’s disease. *Hum. Brain Mapp.* 12 42–60. 10.1002/1097-0193(200101)12:1<42::aid-hbm40>3.0.co;2-d 11198104 PMC6872067

[B62] Neuling T. Rach S. Herrmann C. S. (2013). Orchestrating neuronal networks: sustained after-effects of transcranial alternating current stimulation depend upon brain states. *Front. Hum. Neurosci.* 7:161. 10.3389/fncel.2019.00400 23641206 PMC3639376

[B63] Nitsche M. A. Paulus W. (2000). Excitability changes induced in the human motor cortex by weak transcranial direct current stimulation. *J. Physiol.* 527 633–639. 10.1111/j.1469-7793.2000.t01-1-00633.x 10990547 PMC2270099

[B64] Nonnekes J. Timmer M. H. de Vries N. M. Rascol O. Helmich R. C. Bloem B. R. (2016). Unmasking levodopa resistance in Parkinson’s disease. *Mov. Disord.* 31 1602–1609. 10.1002/mds.26712 27430479

[B65] Nutt J. G. Bloem B. R. Giladi N. Hallett M. Horak F. B. Nieuwboer A. (2011). Freezing of gait: moving forward on a mysterious clinical phenomenon. *Lancet Neurol.* 10 734–744. 10.1016/S1474-4422(11)70143-0 21777828 PMC7293393

[B66] Pereira J. B. Junqué C. Bartrés-Faz D. Martí M. J. Sala-Llonch R. Compta Y. (2013). Modulation of verbal fluency networks by transcranial direct current stimulation (tDCS) in Parkinson’s disease. *Brain Stimul.* 6 16–24. 10.1016/j.brs.2012.01.006 22410476

[B67] Possti D. Fahoum F. Sosnik R. Giladi N. Hausdorff J. M. Mirelman A. (2021). Changes in the EEG spectral power during dual-task walking with aging and Parkinson’s disease: initial findings using Event-Related Spectral Perturbation analysis. *J. Neurol.* 268 161–168. 10.1007/s00415-020-10104-1 32754831

[B68] Powers R. Etezadi-Amoli M. Arnold E. M. Kianian S. Mance I. Gibiansky M. (2021). Smartwatch inertial sensors continuously monitor real-world motor fluctuations in Parkinson’s disease. *Sci. Transl. Med.* 13:eabd7865. 10.1126/scitranslmed.abd7865 33536284

[B69] Rascol O. Sabatini U. Fabre N. Brefel C. Loubinoux I. Celsis P. (1997). The ipsilateral cerebellar hemisphere is overactive during hand movements in akinetic parkinsonian patients. *Brain* 120 103–110. 10.1093/brain/120.1.103 9055801

[B70] Rektorova I. Sedlackova S. Telecka S. Hlubocky A. Rektor I. (2007). Repetitive transcranial stimulation for freezing of gait in Parkinson’s disease. *Mov. Disord.* 22 1518–1519. 17516472 10.1002/mds.21289

[B71] Ridding M. C. Inzelberg R. Rothwell J. C. (1995). Changes in excitability of motor cortical circuitry in patients with Parkinson’s disease. *Ann. Neurol.* 37 181–188. 10.1002/ana.410370208 7847860

[B72] Rosin B. Slovik M. Mitelman R. Rivlin-Etzion M. Haber S. N. Israel Z. (2011). Closed-loop deep brain stimulation is superior in ameliorating parkinsonism. *Neuron* 72 370–384. 10.1016/j.neuron.2011.08.023 22017994

[B73] Ruan X. Li Y. Li E. Xie F. Zhang G. Luo Z. (2020). Impaired Topographical Organization of Functional Brain Networks in Parkinson’s Disease Patients With Freezing of Gait. *Front. Aging Neurosci.* 12:580564. 10.3389/fnagi.2020.580564 33192473 PMC7609969

[B74] Sadler C. M. Kami A. T. Nantel J. Carlsen A. N. (2021). Transcranial direct current stimulation of supplementary motor area improves upper limb kinematics in Parkinson’s disease. *Clin. Neurophysiol.* 132 2907–2915. 10.1016/j.clinph.2021.06.031 34412968

[B75] Sanna A. Fattore L. Badas P. Corona G. Cocco V. Diana M. (2019). Intermittent Theta Burst Stimulation of the Prefrontal Cortex in Cocaine Use Disorder: A Pilot Study. *Front. Neurosci.* 13:765. 10.3389/fnins.2019.00765 PMC6670008 31402851

[B76] Schutter D. J. Hortensius R. (2010). Retinal origin of phosphenes to transcranial alternating current stimulation. *Clin. Neurophysiol.* 121 1080–1084. 10.1016/j.clinph.2009.10.038 20188625

[B77] Shill H. A. Obradov S. Katsnelson Y. Pizinger R. A. (2011). randomized, double-blind trial of transcranial electrostimulation in early Parkinson’s disease. *Mov. Disord.* 26 1477–1480. 10.1002/mds.23591 21538515

[B78] Sidaway B. Anderson J. Danielson G. Martin L. Smith G. (2006). Effects of long-term gait training using visual cues in an individual with Parkinson disease. *Phys. Ther.* 86 186–194. 16445332

[B79] Soikkeli R. Partanen J. Soininen H. Paakkonen A. Riekkinen P. Sr. (1991). Slowing of EEG in Parkinson’s disease. *Electroencephalogr. Clin. Neurophysiol.* 79 159–165. 1714807 10.1016/0013-4694(91)90134-p

[B80] Stephani C. Nitsche M. A. Sommer M. Paulus W. (2011). Impairment of motor cortex plasticity in Parkinson’s disease, as revealed by theta-burst-transcranial magnetic stimulation and transcranial random noise stimulation. *Park. Relat. Dis.* 17 297–298. 10.1016/j.parkreldis.2011.01.006 21316290

[B81] Swank C. Mehta J. Criminger C. (2016). Transcranial direct current stimulation lessens dual task cost in people with Parkinson’s disease. *Neurosci. Lett.* 626 1–5. 10.1016/j.neulet.2016.05.010 27181509

[B82] Swann N. C. de Hemptinne C. Thompson M. C. Miocinovic S. Miller A. M. Gilron R. (2018). Adaptive deep brain stimulation for Parkinson’s disease using motor cortex sensing. *J. Neural. Eng.* 15:046006. 29741160 10.1088/1741-2552/aabc9b PMC6021210

[B83] Terney D. Chaieb L. Moliadze V. Antal A. Paulus W. (2008). Increasing human brain excitability by transcranial high-frequency random noise stimulation. *J. Neurosci.* 28 14147–14155. 10.1523/JNEUROSCI.4248-08.2008 19109497 PMC6671476

[B84] Van Den Heuvel M. P. Pol H. E. H. (2010). Exploring the brain network: a review on resting-state fMRI functional connectivity. *Eur. Neuropsychopharmacol.* 20 519–534. 10.1016/j.euroneuro.2010.03.008 20471808

[B85] Vasquez A. Malavera A. Doruk D. Morales-Quezada L. Carvalho S. Leite J. (2016). Duration Dependent Effects of Transcranial Pulsed Current Stimulation (tPCS) Indexed by Electroencephalography. *Neuromodulation* 19 679–688. 10.1111/ner.12457 27400423

[B86] Wach C. Krause V. Moliadze V. Paulus W. Schnitzler A. Pollok B. (2013). The effect of 10 Hz transcranial alternating current stimulation (tACS) on corticomuscular coherence. *Front. Hum. Neurosci.* 7:511. 10.3389/fnhum.2013.00511 24009573 PMC3756226

[B87] Weber M. J. Messing S. B. Rao H. Detre J. A. Thompson-Schill S. L. (2014). Prefrontal transcranial direct current stimulation alters activation and connectivity in cortical and subcortical reward systems: a tDCS-fMRI study. *Hum. Brain Mapp.* 35 3673–3686. 10.1002/hbm.22429 24453107 PMC4107089

[B88] Wirdefeldt K. Adami H. O. Cole P. Trichopoulos D. Mandel J. (2011). Epidemiology and etiology of Parkinson’s disease: a review of the evidence. *Eur. J. Epidemiol.* 26 S1–S58. 10.1007/s10654-011-9581-6 21626386

[B89] Workman C. D. Fietsam A. C. Uc E. Y. Rudroff T. (2020). Cerebellar transcranial direct current stimulation in people with Parkinson’s disease: a pilot study. *Brain Sci.* 10:96. 10.3390/brainsci10020096 32053889 PMC7071613

[B90] Yaqub M. A. Woo S.-W. Hong K.-S. (2018). Effects of HD-tDCS on resting-state functional connectivity in the prefrontal cortex: an fNIRS study. *Complexity* 2018 1-13

[B91] Yu H. Sternad D. Corcos D. M. Vaillancourt D. E. (2007). Role of hyperactive cerebellum and motor cortex in Parkinson’s disease. *Neuroimage* 35 222–233. 10.1016/j.neuroimage.2006.11.047 17223579 PMC1853309

[B92] Zhang Q. Hu S. Talay R. Xiao Z. Rosenberg D. Liu Y. (2021). A prototype closed-loop brain-machine interface for the study and treatment of pain. *Nat. Biomed. Eng.* 10.1038/s41551-021-00736-7 34155354 PMC9516430

[B93] Ziemann U. Ilic T. V. Jung P. (2006). Long-term potentiation (LTP)-like plasticity and learning in human motor cortex–investigations with transcranial magnetic stimulation (TMS). *Suppl. Clin. Neurophysiol.* 59 19–25. 10.1016/s1567-424x(09)70007-8 16893088

